# Bioinspired bi-phasic 3D nanoflowers of MgO/Mg(OH)_2_ coated melamine sponge as a novel bactericidal agent

**DOI:** 10.1038/s41598-023-40336-w

**Published:** 2023-08-16

**Authors:** Ashutosh Agarwal, Hasanthi L. Senevirathna, Seok Hwee Koo, Crystal Shie Lyeen Wong, Terence Sey Kiat Lim, Foo Cheong Ng, Franklin Anariba, Ping Wu

**Affiliations:** 1 Entropic Interface Group, Engineering Product Development, Singapore University of Technology and Design, 8 Somapah Road, Singapore, 487372 Singapore; 2 Clinical Trials and Research Unit, Changi General Hospital, 2 Simei Street 3, Singapore, 529889 Singapore; 3 Department of Laboratory Medicine, Changi General Hospital, 2 Simei Street 3, Singapore, 529889 Singapore; 4 Department of Urology, Changi General Hospital, 2 Simei Street 3, Singapore, 529889 Singapore; 5 Anariba Brands Group, Science, Mathematics and Technology, Affiliated to Engineering Product Development, Singapore University of Technology and Design, 8 Somapah Road, Singapore, 487372 Singapore

**Keywords:** Biological techniques, Biotechnology, Cell biology, Medical research, Urology, Materials science, Nanoscience and technology

## Abstract

By roughly mimicking the surface architectural design of dragonfly wings, novel bi-phasic 3D nanoflowers of MgO/Mg(OH)_2_ were successfully synthesized via the electrospinning technique. The 3D nanoflowers were coated over a commercial melamine sponge and extensively characterized by SEM, XRD, FTIR, and EDS. The formation of distinct dense 3D nano petals was revealed by SEM images whereby the mean petal thickness and mean distance between the adjacent petals were found to be 36 nm and 121 nm, respectively. The bactericidal activities of synthesized 3D nano-flowers coated melamine sponges were assessed against five different bacteria (*Staphylococcus aureus*, *Enterococcus faecalis, Escherichia coli, Klebsiella pneumoniae, and Pseudomonas aeruginosa*). This study demonstrated significant bactericidal activity of MgO/Mg(OH)_2_ 3D nanoflowers coated MS against Gram-positive and Gram-negative bacteria. Plausible bactericidal mechanisms include envelope deformation, penetration, and induction of oxidative stress. This study introduces novel bioinspired biomaterial with the capacity to reduce the risk associated with pathogenic bacterial infections, especially in medical devices.

## Introduction

Antibacterial surfaces are in high demand for biomedical applications to reduce infections associated with implanted medical devices that costs nearly 5–10 billion dollars a year^1^. Several nanoparticles such as that of titanium oxide, zinc oxide, and silver oxide have shown reasonable bactericidal activity toward a wide spectrum of bacterial strains^2,3^. However, the cytotoxic effects associated with the accumulation of heavy metal elements-based nanoparticles in the human body have raised serious concerns against their use as bactericidal agents^4,5^. Although coating of biomedical devices with biocidal agents such as silver and antibiotics is a conventional biochemical approach; concomitantly, excessive use of antibiotics renders bacteria drug-resistant and causes chronic infections^6^. It has been estimated that nearly 10% of the patients that undergo medical implant surgery develop acute bacterial infections causing an average of 0.1 million deaths in the United States^7^. Once the infection occurs at the biomedical implant-tissue interface, the removal of the medical device and/or implant via secondary surgery becomes inevitable. This not only causes discomfort for the patient but also increases the healthcare cost^8^. With the excessive use of antibiotics, the ever-increasing emergence of antibiotic-resistant bacterial strains has rendered the need to discover novel antibacterial strategies and bactericidal agents^9^.

In the past decade, nano-scale modification of surfaces has offered new routes for the creation of engineered anti-bacterial topographies^10^. For instance, grooves, ridges and ripples-based structures have shown enormous potential in mitigating biofilm formation by reducing bacterial adhesion ^10,11^. Recently, the discovery of biocidal activity of naturally occurring high aspect ratio nano-architectures that appears on the surface of insect wings such as cicadae and dragonflies has triggered the research for novel antibacterial nanomaterial topographies^12^. The physical interactions between the attached pathogens and nanoscale topographies drive the antimicrobial activity of such surfaces^13^. Besides insect wings, other naturally occurring anti-biofouling and self-cleaning surfaces include rice leaves^14^, lotus leaves^15^, gecko skin^16^ and shark skin^17,18^. Inspired by the bactericidal nano-architecture of natural materials, nano protrusions have been successfully developed with titanium^19,20^, gold^21^, diamond^22^, black silicon^23^ and poly(methyl methacrylate)^24^. Almost all of these artificial nano-architectures display excellent biocidal activity towards both Gram-positive and Gram-negative bacteria.

Magnesium-based alloys remain the most desirable bio-implant material owing to their biocompatible and non-toxic nature. Besides being non-toxic in nature, these materials are enriched with marvelous mechanical properties comparable to that of human bone^25^. The biocompatible and non-toxic nature of magnesium-based materials as well as their widespread application in the biomedical field has encouraged us to elect magnesium for the conception of novel bactericidal topographies. In this research, 3D nanoflowers of bi-phasic MgO/Mg(OH)_2_ were successfully synthesized by roughly mimicking the surface architectural design of dragonfly wings. The 3D nanoflowers were synthesized by electrospinning technique followed by calcination and steaming. Analytical techniques such as XRD, SEM, FTIR, and EDS were employed to comprehensively characterize the 3D nanoflowers. With the assumption that the surface topography of 3D nanoflowers would exhibit similar bactericidal activity as that of dragonfly wings, the freshly prepared nanoflowers were incorporated into commercial melamine sponge (MS). MS was adopted as the support material due to its intrinsic properties such as low cost, non-toxic nature, high porosity, highly permeable channels, and 3D architecture that provides large surface area suitable for the deposition of nano materials. The bactericidal efficacies of bi-phasic MgO/Mg(OH)_2_ 3D nanoflowers coated MS were investigated against five different bacteria including both Gram-positive and Gram-negative bacteria (*Staphylococcus aureus*, *Enterococcus faecalis, Escherichia coli, Klebsiella pneumoniae, and Pseudomonas aeruginosa*).

## Experimental section

### Materials

Magnesium hydroxide (reagent grade, 95%) and polyvinyl alcohol (PVA) (87–90% hydrolyzed, molecular weight 30,000–70,000), were purchased from Sigma-Aldrich while glacial acetic acid (analytical grade, 99.8%), polyacrylic acid (PAA) (25 wt% in water), and chitosan were acquired from Scharlau, Alfa Aesar, and MP Biomedicals, respectively. Melamine sponge (MS) (brand: Vesta) was sourced from a local e-commerce company. All the chemicals were used as received without further purification.

### Methods

#### *Synthesis of 3D nanoflowers of MgO/Mg(OH)*_*2*_

0.25 g Mg(OH)_2_ was dissolved in 5 ml glacial acetic acid at 55 °C for 1 h in an ultrasonication bath (brand: Elmasonic P, 37 kHz). An aqueous PVA solution (5% w/w) was prepared by dissolving PVA powder in deionized (DI) water at room temperature under continuous magnetic stirring for 12 h at 600 rpm. Both the above solutions were mixed in a ratio of 15:100 (v:v) and ultrasonicated (37 kHz) at 55 °C for 20 min to obtain a clear solution. This solution was then placed in a 5 ml Terumo® syringe equipped with a 21Gx1/2″ gauge-size needle. Electrospinning was carried out with a needle-collector top-down configuration whereby the distance between the needle and the collector was maintained at 13 cm. The solution flow rate and applied DC voltage were set at 0.3 ml h^−1^ and 17 kV, respectively. The nanofiber layers were deposited on Al foil spread across the collector. In the end, the sample collected over Al foil was dried in a hot air oven at 110 °C for 24 h to obtain a brittle layer. The flakes collected after scratching this brittle layer were calcined in a muffle furnace (Nabertherm, Germany) at 350 °C for 1 h. The heating rate of the furnace was set at 2 °C min^-1^. After calcination, the samples were allowed to cool down naturally to room temperature and then ground to a fine powder using a mortar and pestle. Finally, the fine powder was subjected to steam for 40 min in a steam oven (Toshiba). After steaming, the powder was collected and dried at room temperature in a vacuum desiccator for 24 h.

#### *Coating 3D nanoflowers of MgO/Mg(OH)*_*2*_* over melamine sponge*

The commercial MS was cut into a cuboid (1 cm × 1 cm × 0.5 cm) with the help of an art knife and soaked in freshly prepared 0.1 wt% PAA aqueous solution (pH = 1, adjusted with 1 M HCl) for 5 min. The MS was thoroughly squeezed with a pair of tweezers and then soaked in 0.5 wt% chitosan aqueous solution (pH = 5, adjusted with 1 M HCl) for another 5 min followed by sequential squeezing multiple times during the entire process. Through this process, PAA and chitosan bind together by electrostatic interaction^26^ and provide active sites for the deposition of nanoflowers. The MS was washed with DI water and finally immersed in 0.3 wt% aqueous suspensions of MgO/Mg(OH)_2_ 3D nanoflowers for 20 min. During this process, MS was squeezed with a pair of tweezers and re-soaked in the aqueous suspension after 10 min. The 3D nanoflowers coated MS thus obtained was dried in a hot air oven at 60 °C for 6 h.

### Characterization techniques

X-ray diffraction (XRD) diffractograms of biphasic MgO/Mg(OH)_2_ were recorded with Bruker D8 Advance X-ray diffractometer equipped with LYNXEYE detector. The X-ray generator was operating at 40 kV and 25 mA. The diffractograms were recorded at angles (*2θ*) between 10° and 70° with a step size of 0.03° (*2θ*) employing Cu K_α_ radiation (λ = 1.5406 Å). The crystallite size (*d*) of MgO/Mg(OH)_2_ nanoparticles was evaluated by Debye–Scherrer’s formula (Eq. 1).

1 $$d=\frac{0.9 \lambda }{\beta \mathrm{cos}\theta }$$

where 0.9 represents the molecular shape factor, λ is the wavelength of Cu K_α_ radiation (1.5406 Å), β is the full width at half maxima (radians) and θ is the Bragg’s diffraction angle (radians) corresponding to the most intense peak.

Surface morphologies of the samples were examined by scanning electron microscope (SEM) (JEOL JSM-7600 F). The sample imaging was improved by sputter coating with Au. Sputter coating enriches the secondary electron signal required for topographic examination by inhibiting sample charging during SEM analysis. ImageJ software (version 1.53t) from the National Institute of Health, USA was used to analyze the SEM images. The elemental imaging of coated melamine sponge was carried out by energy-dispersive spectroscopy (EDS) (X-MaxN-50, Oxford Instruments) embedded within the SEM. Fourier transform infrared spectroscopy (FTIR) was conducted to determine the chemical structure of the samples. For this purpose, Agilent Cary 630 FTIR spectrometer was employed to collect FTIR spectra at a resolution of 1 cm^−1^ between the wavelengths 650–4000 cm^−1^ with 32 scans each.

### Antibacterial tests

Five different bacteria strains (*S. aureus, E. faecalis, E. coli, K. pneumoniae, and P. aeruginosa*) were used to ascertain the bactericidal effects of 3D nanoflowers coated MS. Each bacteria was sub-cultured onto trypticase soy agar (TSA) with 5% sheep blood plate and incubated for 18–24 h before commencing the antibacterial study. A 0.5 McFarland bacterial suspension was prepared for each organism using a sterile saline solution. Each MS weighing between 0.021 and 0.032 g was fully immersed in defined volumes between 1.68 and 2.56 ml (0.0125 g of MS per ml) of bacterial suspension with a concentration of 10^4^–10^5^ cfu ml^−1^ and incubated for a total of 24 h. From each bacterial suspension a sampling volume of 10 ul was collected at baseline (t = 0) and defined intervals (t = 2, 4, 8, and 24 h) of incubation and plated onto culture plates in duplicates, which were then incubated for 18–24 h before enumeration. All incubation was performed at 35 °C, under aerobic conditions. The presence of any bacterial growth on the culture plates was enumerated and the average colony-forming unit (cfu) of the two plates was calculated for each bacteria. A control suspension (bacteria only without the MS) was set up alongside each corresponding test suspension and sampled as per the time intervals above. Another control solution (sterile saline with MS) was also set up and sampled at t = 0 and t = 24 h to control for sterility throughout the study.

## Results and discussion

**Figure 1:**
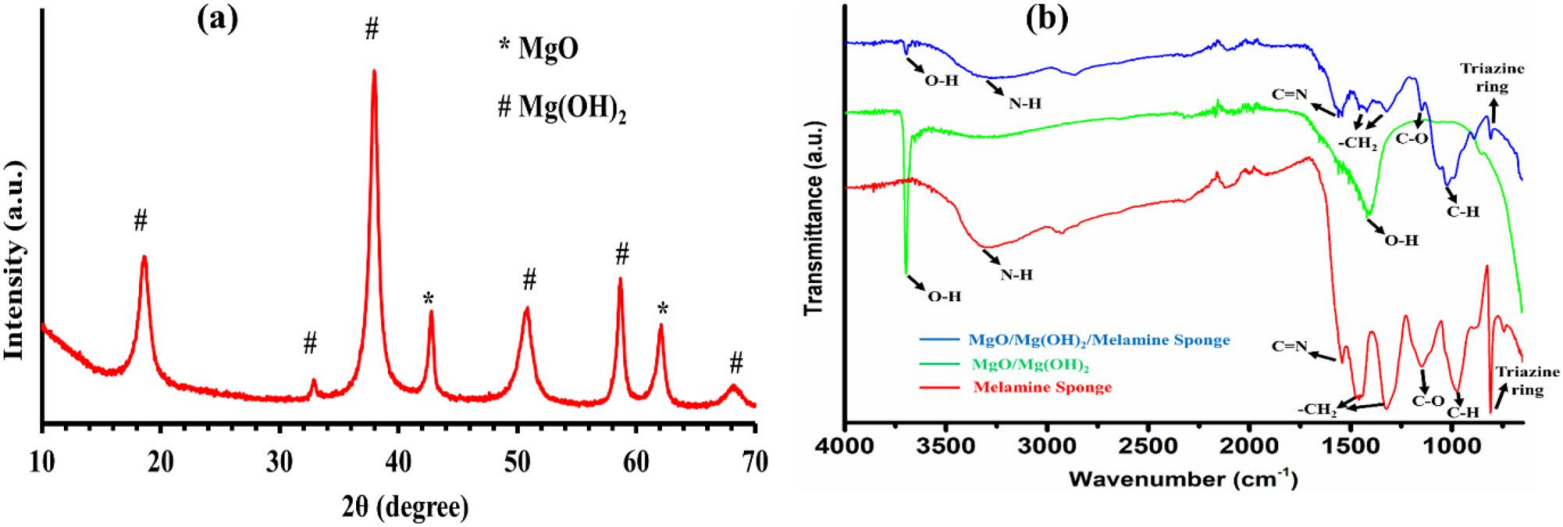
(**a**) Powder XRD spectrum of biphasic MgO/Mg(OH)_2_; (**b**) FTIR spectra of uncoated melamine sponge, MgO/Mg(OH)_2_ and MgO/Mg(OH)_2_ coated melamine sponge.

Figure 1a shows the powder XRD spectrum of biphasic MgO/Mg(OH)_2_ prepared via an electrospinning technique followed by calcination and steaming processes. The peaks at 2θ values 18.5°, 33.0°, 37.9°, 50.7°, 58.6° and 68.1° correspond to (001), (100), (101), (102), (110) and (103) lattice planes of Mg(OH)_2_ (ICDD 00-044-1482) while the two peaks at 2θ values 42.9° and 62.3° refers to (200) and (220) lattice planes of MgO (ICDD 00-045-0946), respectively. The existence of diffraction peaks corresponding to both MgO and Mg(OH)_2_ explicitly confirms the formation of biphasic MgO/Mg(OH)_2_. The crystallite size as calculated from the most intense peak (37.9°) was found to be 8.8 nm. In addition, the high crystallinity of the synthesized nanomaterial was apparent from the presence of intense XRD peaks.

The FTIR spectra of MgO/Mg(OH)_2_ 3D nanoflowers, uncoated MS, and MgO/Mg(OH)_2_ 3D nanoflowers coated MS are shown in Fig. 1b. The spectrum of biphasic MgO/Mg(OH)_2_ displays sharp intense peak at 3697 cm^−1^ and a distinct band at 1411 cm^−1^. These were ascribed to the stretching (3697 cm^−1^) and bending (1411 cm^−1^) vibrations of the surface hydroxyl group^27^. In the FTIR spectra of MgO/Mg(OH)_2_ coated and uncoated MS, the peaks at 3312, 1559, 1460, 1313, 1145, 1013, 806 cm^-1^ refer to the N–H stretching, C=N stretching, –CH_2_– bending, C–O stretching, C–H bending and triazine ring bending vibrations, respectively. The presence of a sharp O–H stretching peak at 3697 cm^-1^ in the FTIR spectrum of MgO/Mg(OH)_2_ 3D nanoflowers coated MS confirms the successful attachment of MgO/Mg(OH)_2_ to the MS.

**Figure 2:**
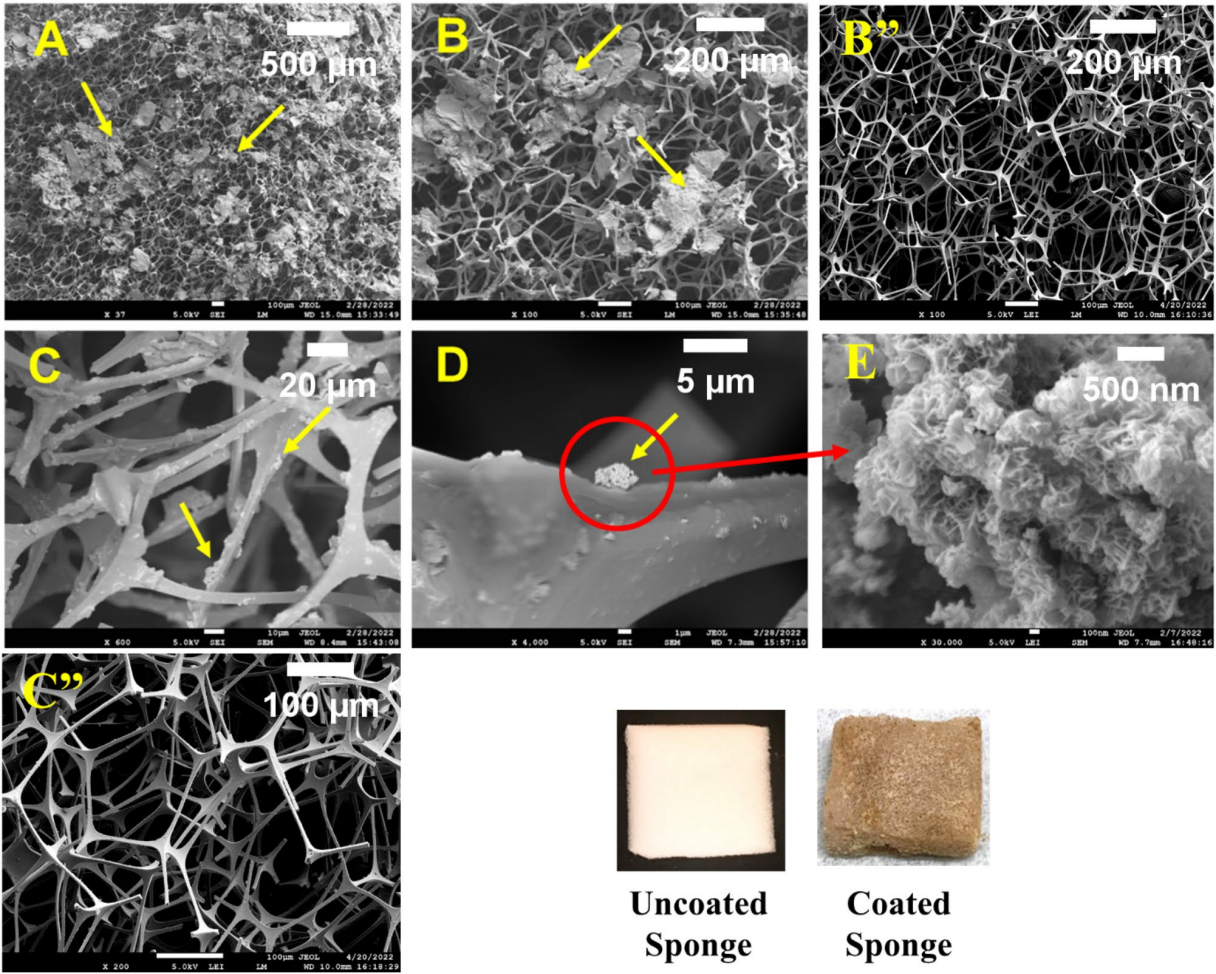
SEM images of uncoated melamine sponge (**B**”, **C**”), MgO/Mg(OH)_2_ coated melamine sponge (**A**, **B**, **C**, **D**) at different magnifications and MgO/Mg(OH)_2_ 3D nanoflowers showing distinct petals (**E**).

**Figure 3:**
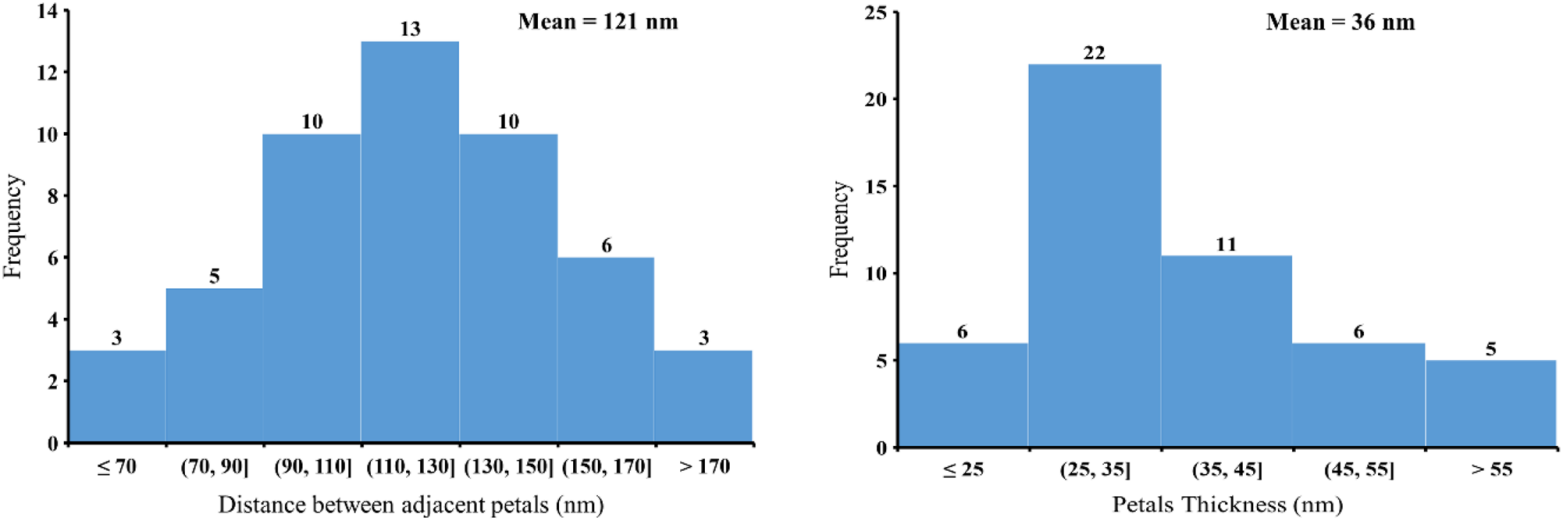
Histogram plots of distance between adjacent petals and petals thickness.

**Figure 4:**
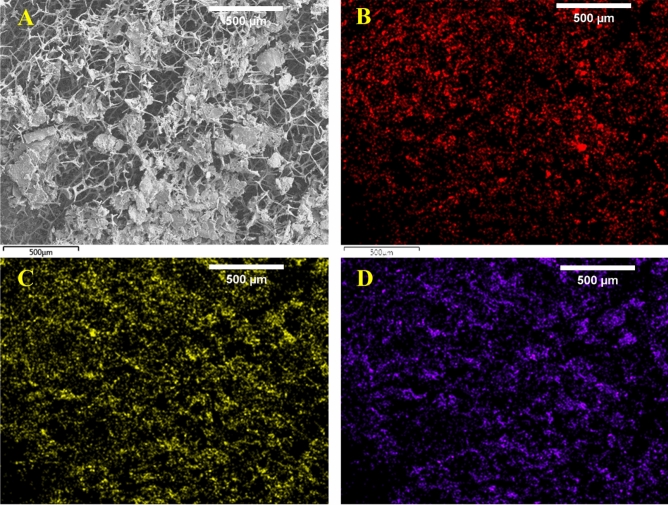
SEM image (**A**) and EDS mapping of the cross-sectional view of MgO/Mg(OH)_2_ coated melamine sponge showing distribution of magnesium (**B**), carbon (**C**) and oxygen (**D**).

The morphologies of MgO/Mg(OH)_2_ 3D nanoflowers, uncoated MS, and MgO/Mg(OH)_2_ 3D nanoflowers coated MS are shown in SEM images (Fig. 2). The uncoated MS exhibit highly inerratic 3D reticular structure with pore size ranging between 100 and 200 µm (Fig. 2B” and C”). The deposition of MgO/Mg(OH)_2_ (marked by arrows) onto the MS skeleton is visible in SEM images (Fig. 2A,B,C,D). After coating with 3D nanoflowers of MgO/Mg(OH)_2_, the smooth MS surface became rough while the intrinsic 3D reticular structure of MS remained almost intact. Subsequently, the color of MS changed from pure white to light brown. The magnified SEM image (Fig. 2E) of MgO/Mg(OH)_2_ 3D nanoflowers coated MS revealed the formation of distinct dense nano petals. Upon analysis with image analysis software (ImageJ), the mean petal thickness and mean distance between adjacent petals of 3D nanoflowers were found to be 36 nm and 121 nm, respectively. Both these parameters were determined using a sample size of 50. From the histogram plots (Fig. 3), it is obvious that the adjacent distance between the majority of 3D nanoflower petals ranges between 90 and 130 nm while the petal’s thickness varies from 25 to 45 nm. Figure 4 shows EDS mapping of the cross-sectional view of MgO/Mg(OH)_2_ coated MS. The major elements vis. Mg, C, and O were uniformly distributed over the entire subject area. This evidence confirms that the 3D nanoflowers of MgO/Mg(OH)_2_ were incorporated deep inside the inerratic 3D reticular structure of the MS. Indeed, the uniform light brown color of MgO/Mg(OH)_2_ coated MS ascertains homogeneous distribution of MgO/Mg(OH)_2_ nanoflowers throughout the 3D network of MS.

**Figure 5:**
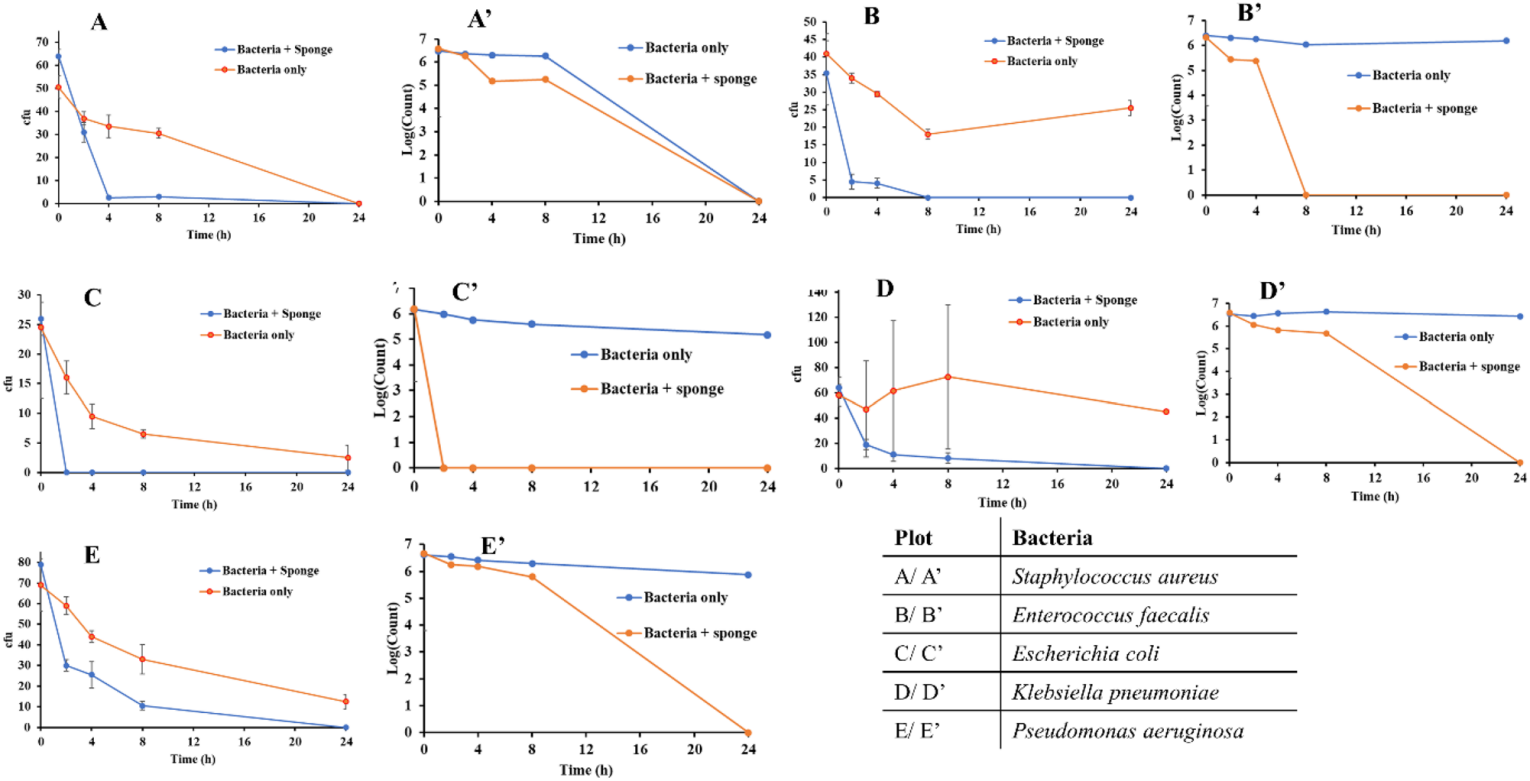
Temporal variation in cfu count and Log(count) of *Staphylococcus aureus* (**A**/**A**’),* Enterococcus faecalis* (**B**/**B**’),* Escherichia coli* (**C**/**C**’),* Klebsiella pneumoniae* (**D**/**D**’) and *Pseudomonas aeruginosa* (**E**/**E**’) with and without MgO/Mg(OH)_2_ 3D nanoflowers coated melamine sponge. Error bars are 1 SD from the mean of triplicate measurements.

The bactericidal efficacy of MgO/Mg(OH)_2_ 3D nanoflowers coated MS was determined over a period of 24 h against five different bacteria including two Gram-positive (*S. aureus* and *E. faecalis*) and three Gram-negative (*E. coli, K. pneumoniae,* and *P. aeruginosa*) bacteria. Figure 5 shows temporal variation in cfu and Log(count) of *S. aureus*, *E. faecalis*, *E. coli*, *K. pneumoniae,* and *P. aeruginosa* upon exposure to MgO/Mg(OH)_2_ 3D nanoflowers coated MS. In this research, the selected Gram-positive and Gram-negative bacteria were spherical-shaped and rod-shaped, respectively. The initial 2 h bactericidal rate of 3D nanoflowers coated MS follows the sequence: *P. aeruginosa* > *K. pneumoniae* > *S. aureus* > *E. faecalis* (Fig. 5). From this trend, it is evident that in general the initial rate of bactericidal activity was higher for rod-shaped than spherical-shaped bacteria. This was attributed to the large surface area of rod-shaped (Gram-negative) bacteria in direct contact with nano petals than spherical-shaped (Gram-positive) bacteria. In addition, thick and rigid cell walls of Gram-positive (spherical-shaped) bacteria allow them to withstand large mechanical deformation compared to Gram-negative bacteria (rod-shaped) which usually comprise thin and fragile cell walls^28^. On this fact, the nanopillar structure of *Psaltoda claripennis* cicada wings also shows bactericidal activity only against Gram-negative bacterial strains^29^.

**Figure 6:**
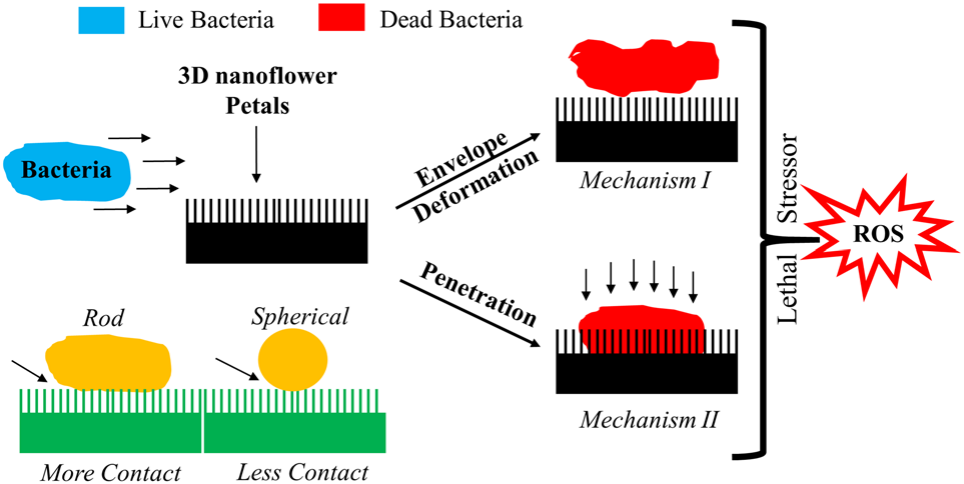
Schematic illustration of bactericidal mechanism of MgO/Mg(OH)_2_ 3D nanoflowers coated melamine sponge.

Figure S1 shows the release profile of Mg from MgO/Mg(OH)_2_ coated melamine sponge at 35 °C. Since only an infinitesimal fraction of Mg was released over a duration of 24 h, it is apparent that the bactericidal activity was unassociated with the release of Mg from the MgO/Mg(OH)_2_ coated sponge. A plausible schematic illustration of the bactericidal mechanisms of 3D nano petals of MgO/Mg(OH)_2_ coated melamine sponge is shown in Fig. 6. Since the mean distance (121 nm) between the adjacent petals of MgO/Mg(OH)_2_ 3D nanoflowers is much smaller than the minimum dimension (250 nm) of the bacteria; it is quite obvious that bacteria could easily rest over the nano petals array without tumbling into the gorge between them. Considering that the petals are substantially thinner (36 nm); it is expected that as the bacteria are pulled down towards the surface by adhesive and/or gravitational forces, the bacterial envelope is more likely to be deformed and penetrated by the nailing action of rigid nano petals *(Mechanism I & II)*^30^. Nano protrusions-induced bacterial envelope penetration and deformation have been well ascertained by bacterial cross-sectional analysis employing transmission electron microscopy (TEM), focused ion beam (FIB) microscopy, and tomography^31^. Strong experimental evidence suggests that Gram-negative bacteria are more prone to bacterial envelope deformation and penetration than Gram-positive bacteria^31^. Gram-positive bacteria’s reduced vulnerability towards nano protrusions deformation is mainly due to thick peptidoglycan layers that provide high rigidity and turgor pressure^29^. Notably, there is no experimental evidence in the literature that could advocate nano protrusions-induced mechanical rupture or lysis of bacterial cells. According to contact mechanic analysis (Hertzian model), the intrinsic pressure on a single nano protrusion in contact with a bacterium increases with a decrease in tip diameter. For instance, as the nano protrusion tip diameter decreases from 80 nm (blunt) to 40 nm (sharp), the corresponding intrinsic pressure increases from 3.7 to ~ 7.1 MPa^32^. Real-time imaging of mechanically-induced bacterial death by simultaneous nanoindentation and fluorescence microscopy validates that a pressure greater than 10.7 MPa is essential to puncture the cell wall of *E.coli*^33^. Since in our case, the mean petal’s thickness was 36 nm, the intrinsic pressure generated by nano petals in contact with the bacterium was much less than the minimum pressure required to puncture the bacterial cell wall. Although the pressure generated by a single nanoprotrusion tip of diameter 40 nm is insufficient to mechanically rupture the bacterial envelope; the increase in nanoprotrusions density however increases the bacterial cell membrane permeability due to high stretching of the suspended envelope. This fact has been experimentally verified elsewhere by comparison of LIVE/DEAD and BacTiter Glo assay data of Gram-positive and Gram-negative bacteria subject to surfaces with wide and dense nanopillars^32^. Hence, an optimal spacing between adjacent nano petals is highly desirable to achieve adequate bactericidal activity. For instance, a low pitch of interspacing (~ 100 nm) drastically improves the bactericidal efficiency of silicon nanostructures^34^. Computational analysis based on the finite element method suggests that smaller pillar radii and spacing amplifies envelope deformation about nano protrusion tips and deliver significant in-plane strains.^35^ Simulation studies also reinforce the fact that reduced interspacing boosts bactericidal activity of nano protrusion surfaces^35^. In our research, the mean distance of 121 nm between the adjacent petals of MgO/Mg(OH)_2_ 3D nanoflowers was found to be adequate enough to display substantial bactericidal activity.

Certainly, dense and sharp nano protrusions promote bacterial cell envelope deformation, penetration, and membrane permeability. Bacterial cells subjected to lethal stressors are induced by oxidative stress that triggers the formation of reactive oxygen species (ROS)^36^. Although several protective enzymes such as catalases, dismutase, etc. could detoxify low levels of ROS, if the ROS level crosses a certain threshold limit, bacterial death becomes inevitable and irreversible even after the initial stressor has been detached.^37^ ROS-mediated bacterial cell death has been well documented for Carbon nanotubes (CNTs)^38^. It is known that CNTs impose oxidative stress in both Gram-positive and Gram-negative bacteria by mechanically wrapping around them. This promotes production of ROS leading to bactericidal activity. Compelling experimental evidence favoring induced oxidative stress within bacterial cells upon contact with TiO_2_ nanopillars also corroborate the role of ROS in mediating reduced cell viability^31^.

## Conclusions

The 3D nanoflowers of biphasic MgO/Mg(OH)_2_ synthesized via electrospinning were successfully incorporated into commercial MS. After coating, the intrinsic 3D reticular structure of MS remained almost intact. The adjacent distance between the majorities of 3D nanoflower petals ranged between 90 and 130 nm while the petal’s thickness varied from 25 to 45 nm. EDS mapping of the cross-sectional view of MgO/Mg(OH)_2_ coated MS confirmed the incorporation of 3D nanoflowers of MgO/Mg(OH)_2_ deep inside the inerratic 3D reticular structure of MS. This study demonstrated significant bactericidal activity of MgO/Mg(OH)_2_ 3D nanoflowers coated MS against both Gram-positive and Gram-negative bacteria. It is expected that the novel bioinspired biphasic MgO/Mg(OH)_2_ 3D nanoflowers coated MS could be applied to reduce the risk associated with pathogenic bacterial infections in the medical field.

### Supplementary Information

Supplementary Figure S1.

## Data Availability

The datasets used and/or analyzed during the current study are available from the corresponding author upon reasonable request.
