# Supplementary material for: Bioinspired bi-phasic 3D nanoflowers of MgO/Mg(OH)2 coated melamine sponge as a novel bactericidal agent
Source: Sci Rep. 2023 Aug 16;13:13290. doi: 10.1038/s41598-023-40336-w (PMC10432489; doi:10.1038/s41598-023-40336-w)

**Supplementary Information**

**For**

**Bioinspired bi-phasic 3D nanoflowers of MgO/Mg(OH)_2_ coated melamine sponge as a novel bactericidal agent**

Ashutosh Agarwal^a^, Hasanthi L. Senevirathna^a^, Seok Hwee Koo^b^, Crystal Wong Shie Lyeen^c^, Terence Lim Sey Kiat^d^, Ng Foo Cheong^d^, Franklin Anariba^a,e^, Ping Wu^a*^

^a^Entropic Interface Group, Engineering Product Development, Singapore University of Technology and Design, 8 Somapah Road, 487372, Singapore

^b^Clinical Trials and Research Unit, Changi General Hospital, 2 Simei Street 3, Singapore 529889

^c^Department of Laboratory Medicine, Changi General Hospital, 2 Simei Street 3, Singapore 529889

^d^Department of Urology, Changi General Hospital, 2 Simei Street 3, Singapore 529889

^e^Anariba Brands Group, Science, Mathematics and Technology, affiliated to Engineering Product Development, Singapore University of Technology and Design, 8 Somapah Road, 487372, Singapore

Corresponding Authors:

^*^E-mail: [wuping@sutd.edu.sg](mailto:wuping@sutd.edu.sg)

**Fig. S1** Magnesium release profile of MgO/Mg(OH)_2_ coated melamine sponge.


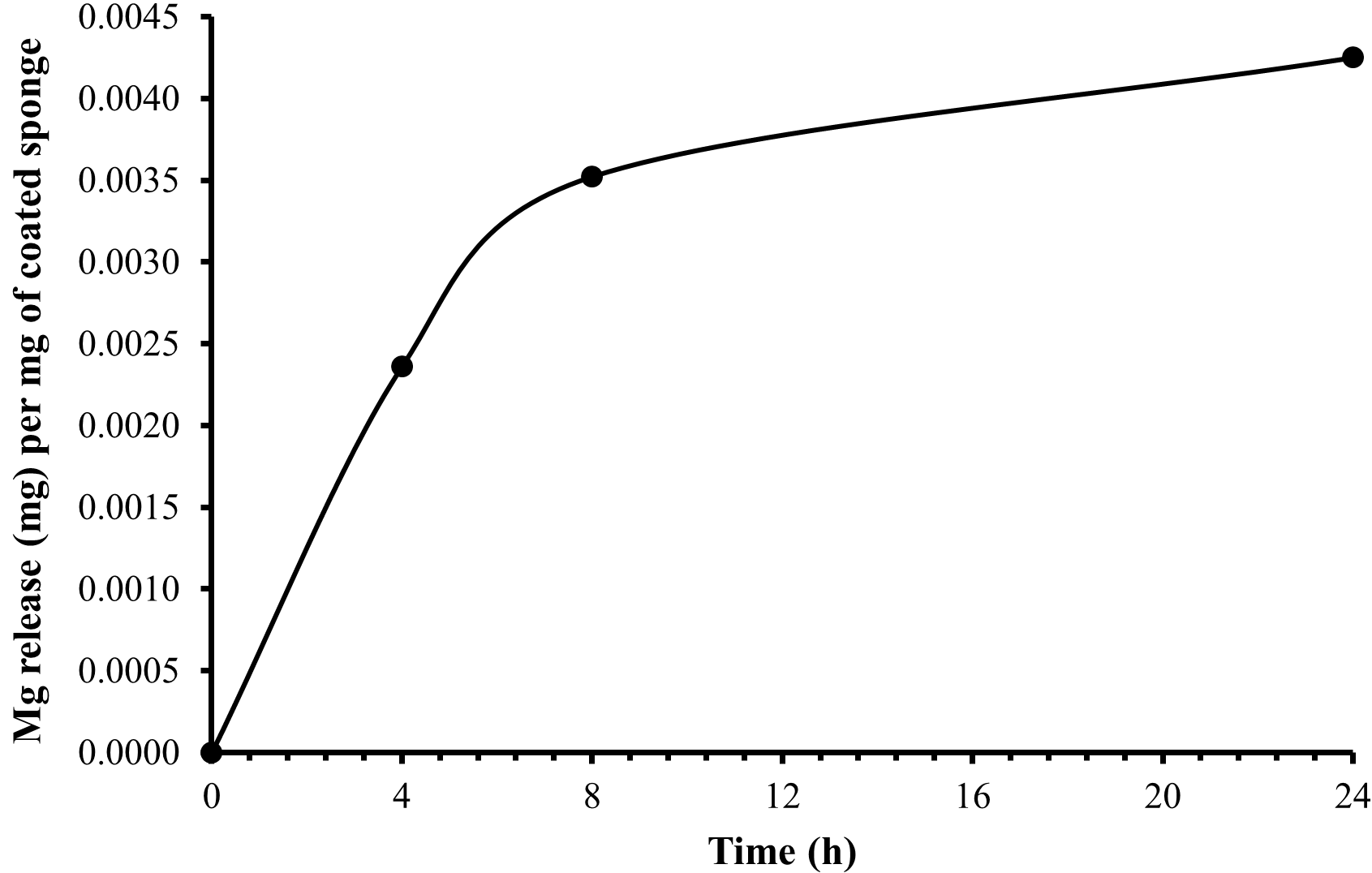

Supplement: Supplementary file 1 — Supplementary Figure S1. [file 41598_2023_40336_MOESM1_ESM.docx]
